# Remote active surveillance following tibial plateau leveling osteotomy: a preliminary study of patellar tendinitis in dogs

**DOI:** 10.3389/fvets.2026.1800916

**Published:** 2026-08-10

**Authors:** Matt Matiasovic, Paula J. Hill, Steve R. Bright, Anna M. Condon, Ben J. Keeley, Karol Bayer

**Affiliations:** 1 Manchester Veterinary Specialists, Worsley, United Kingdom; 2 mmatiasovic.vet Kleintierchirurgie, Delémont, Switzerland; 3 Upskill.vet Veterinary Education, Bratislava, Slovakia; 4 Tierklinik Zürich Ost, Dietlikon, Switzerland

**Keywords:** active surveillance, lameness, patellar tendinitis, TPLO, video-assessment

## Abstract

**Introduction:**

Traditional follow-up methods in small animal orthopedic surgery are primarily passive. These methods have been shown to result in only moderate owner adherence, and their value has been questioned in recent years. In human medicine, active surveillance has been demonstrated to consistently outperform passive methods in detecting adverse events. Our goal was to obtain preliminary data on the utility of active surveillance after tibial plateau leveling osteotomy (TPLO) for detecting patellar tendinitis (PT) associated with lameness and/or pain in dogs.

**Methods:**

Two groups of dogs were retrospectively evaluated for the occurrence of PT. Group 1 (*N* = 910) consisted of a review of data from dogs that underwent routine TPLO follow-up visits, which included history taking, examination, and radiographic assessment. Follow-up for dogs in Group 2 (*N* = 195) was initially performed remotely through active surveillance. Dogs with lameness and/or owner-reported problems were invited for a physical evaluation, which was conducted using the same protocol as in Group 1.

**Results:**

The incidence of PT was 2% (18/910) in Group 1 and 6% (12/195) in Group 2. Owner adherence to follow-up with active surveillance was 85%.

**Discussion:**

Preliminary evidence suggests that remote active surveillance after TPLO may enable reliable detection of clinically relevant PT in dogs. Incorporating structured remote follow-up could improve postoperative monitoring while maintaining diagnostic sensitivity. The limitations of this study include the potential incomplete capture of PT cases in Group 1, the absence of data on activity restriction compliance, and the lack of a demographic comparison between the groups. Further prospective studies are needed to validate this novel approach.

## Introduction

Traditional methods of follow-up investigations—particularly the obtaining of radiographs after elective orthopedic procedures such as TPLO or medial patellar luxation—have been challenged in recent years (1–3). Furthermore, adherence to recommended follow-up appointments after orthopedic surgery is moderate; the authors of one study found that only 66% of dog and cat owners voluntarily attended recommended follow-up visits (4). An alternative to traditional, passive methods of follow-up is active surveillance. Evidence from human and veterinary medicine suggests that active surveillance consistently detects more adverse events—particularly surgical site infections—than passive surveillance (5–7). Active surveillance requires scheduled, purposeful, and separate collection of postoperative data from clients or referring veterinarians and involves review of telephone calls or questionnaires (7, 8).

Radiographic or ultrasonographic thickening of the patellar tendon is a common finding after tibial plateau leveling osteotomy (TPLO), occurring in 48–100% of dogs (3, 9–11). There is limited evidence documenting the clinical relevance (i.e., the occurrence of lameness and/or pain) associated with patellar tendon thickening, which has previously been specifically evaluated in only one study. The authors of that study have found that clinically relevant patellar tendinitis occurred in 7.4% of 94 dogs after TPLO (10). One recent study reported that 2 of 100 TPLO cases were affected by lameness associated with patellar tendinitis (3). The terms tendinitis, desmitis, tendinopathy, and tendinosis are used interchangeably in previous veterinary reports investigating or describing thickening of the patellar tendon post-TPLO with or without associated lameness (1, 3, 10, 12–14). The terms patellar tendon thickening (PTT), referring to radiographic and/or palpatory patellar tendon thickening, and patellar tendinitis (PT), referring to patellar tendon thickening associated with clinical lameness or pain on palpation or stifle manipulation, are used throughout this manuscript.

The recent coronavirus pandemic temporarily reshaped veterinary and medical clinical practice. Remote measures were put in place, including remote follow-up consultations (15–20). Building on previous evidence (1), one such measure adopted in our practice for dogs undergoing TPLO was screening via remote active surveillance (RAS), including gait assessment via video and a targeted telephone interview of owners to determine whether further recovery recommendations or a physical follow-up visit were required.

This study aimed (1) to report the incidence of PT in a previously studied large cohort of routinely assessed dogs after TPLO and (2) to conduct a preliminary assessment of the incidence of PT in a new cohort of dogs after TPLO that were initially screened by RAS. We hypothesized that, with RAS, the detected incidence of PT would be within the known range of 2–7.4%.

## Materials and methods

**Figure 1:**
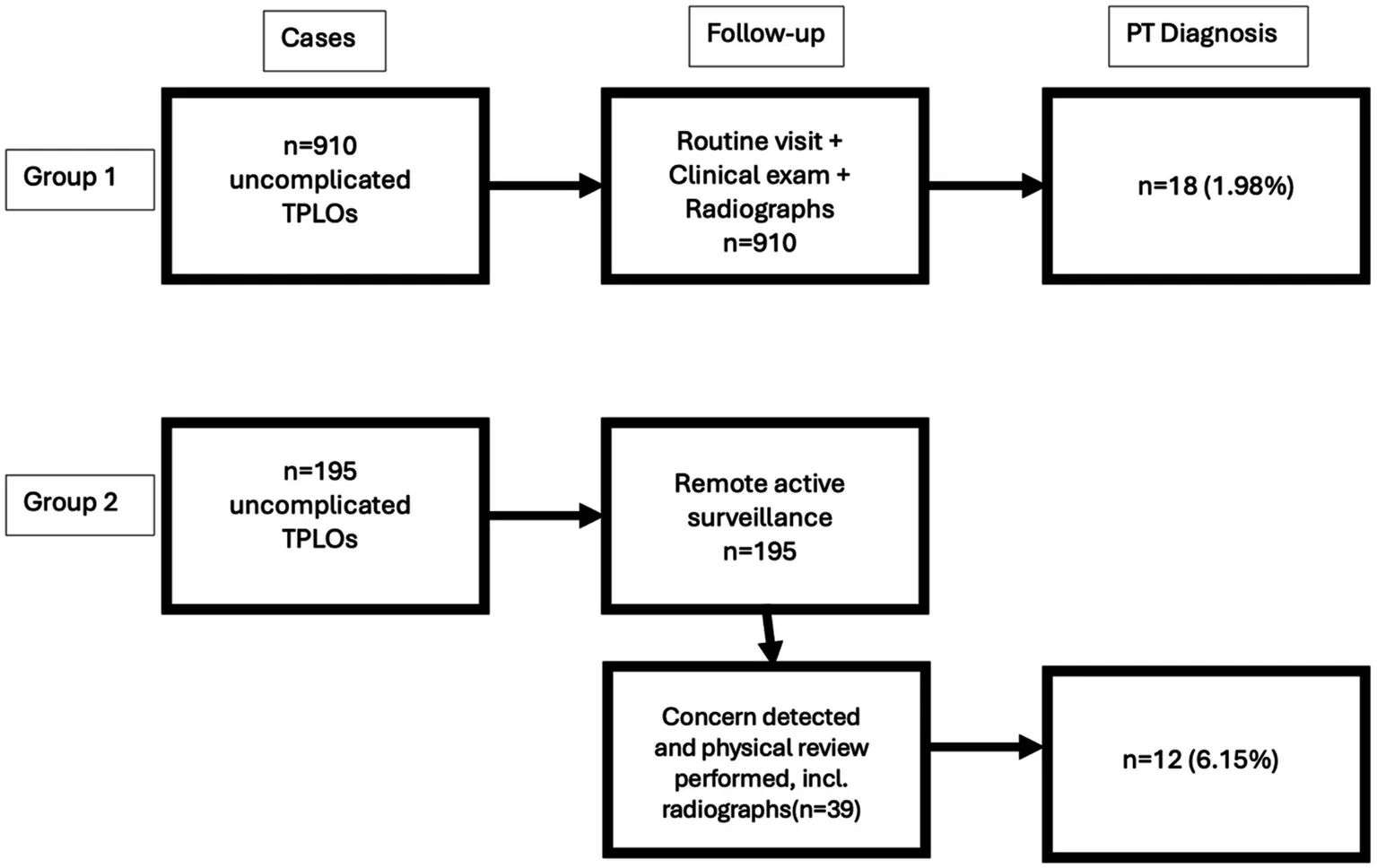
Flowchart navigating two groups of cases of uncomplicated TPLO with a diagnosis of patellar tendinitis that underwent routine in-clinic follow-up or remote active surveillance.

Two groups of dogs that underwent TPLO with subsequent follow-up were investigated (Figure 1). Only surgeries performed by or under the supervision of a recognized specialist in small animal surgery (from the American College of Veterinary Surgeons, European College of Veterinary Surgeons, or Royal College of Veterinary Surgeons) were considered.

*Group 1*: The dataset from a previous study investigating dogs that were presented for routine follow-up, which was planned at approximately 6 weeks postoperatively, when examination and radiographs were performed after uncomplicated TPLO, was analyzed (1). Records of dogs that underwent uncomplicated TPLO prior to April 2020 from 11 institutions were retrospectively reviewed. Requests were sent to participating institutions to evaluate records in reverse chronological order for the dogs that had undergone the 100 most recent uncomplicated TPLO procedures, including routine follow-up clinical and radiographic examination under sedation or general anesthesia. Data from dogs in whom the management plan was altered based on the follow-up visit were evaluated for the presence of a record of lameness, pain on patellar tendon palpation, and radiographic PTT. Uncomplicated TPLO was briefly defined as a unilateral TPLO procedure to treat cranial cruciate ligament disease with no intraoperative complications and no record of surgical site infection or lack of improvement/worsening of the lameness in the period leading to the follow-up event (1). Patellar tendinitis at the follow-up examination was defined as a record of lameness reported by the owner and/or detected by the examining veterinarian with a concurrent record of evidence of PTT on radiography and no other documented cause for the lameness. Cases with incomplete records were excluded.

After the incidence of PT cases in Group 1 was determined, a power analysis was performed using G*Power 3.1.9.7, which revealed that a sample size of 88 dogs was necessary in Group 2 to compare the resulting incidence with that of Group 1, with a medium effect size (*w* = 0.3), an alpha of 0.05, and power (1 − *β*) = 0.80. The goal was then to identify the records of the most recent 50 cases of uncomplicated TPLO performed by each of the surgeons (*n* = 4) at the institution conducting the study between 2021 and 2022. 

*Group 2*: Records of dogs that underwent uncomplicated TPLO prior to July 2023 from a single referral institution were retrospectively reviewed. Records of dogs were reviewed in reverse chronological order for TPLO procedures performed by each of the four surgeons at the institution. TPLO follow-up was routinely performed by RAS (i.e., every dog owner was contacted actively by a single veterinary nurse using a standardized protocol), with follow-up telephone calls targeted at approximately 6 weeks postoperatively. This consisted of obtaining information on the current status of the dog by telephone interview, with questions including the presence of lameness, the level of allowed activity, current administration of medication, the progression of wound healing, and the presence of any owner concerns. Owners were asked to submit video recordings of each dog at the same time. If lameness or other gait abnormalities were visible on video review, or if there were any concerns voiced by the owner, owners were invited to present their dogs to the institution for a physical follow-up examination. Subsequently, if lameness or discomfort of the operated limb was detected on examination, radiographic examinations were performed under sedation. Patellar tendinitis at the follow-up examination was defined as a record of lameness reported by the owner and/or detected by the examining veterinarian and a concurrent record of radiographic evidence of PTT, without evidence of another cause of lameness.

The incidence of PT was compared between the two groups using the Chi-squared test (*χ*^2^ (D*F* = 1) = 10.58, *p* < 0.001). In addition, a bootstrapping procedure was performed to determine the 95% confidence interval for the proportion of PT diagnoses of Group 2. SPSS™ version 30 was used for statistical analysis.

## Results

*Group 1*: Data of 1,010 cases were available for analysis. One hundred cases were excluded because data on the case history at the time of follow-up were lacking. Therefore, 910 cases of uncomplicated TPLO with records of history, clinical examination, and radiography findings at the time of TPLO follow-up were analyzed. The median time from surgery to the scheduled follow-up examination, including radiography, was 6 weeks (range, 4–15). Patellar tendinitis was identified in 18 of 910 cases (1.98%). Cases were categorized into three subgroups based on the combination of owner-reported, clinical, and radiographic findings; full details are summarized in Table 1.

**Table 1:** Findings at the time of follow-up for two groups of dogs with a diagnosis of patellar tendinitis associated with lameness and/or pain on palpation or stifle.

Patellar tendinitis	Group 1 (*n* = 18)			Group 2 (*n* = 12)		
Abnormality	Owner reported	Clinical examination	Radiographic	Owner reported	Clinical examination	Radiographic
Lameness	8	16		4	12	
Discomfort	2	6				
Stiffness	1	1		4		
Off loading in stance				1		
Stifle thickening		5				
Persistent mild tibial thrust		1				
Muscle atrophy		1			2	
Reduced stifle ROM		1				
PTT		2	18			12
Delayed osteotomy healing			5			1
Loss of reduction			2			
Healing TT fracture above patellar tendon insertion			1			

Group 1 underwent standard scheduled follow-up. Group 2 was followed up by active surveillance. ROM, range of motion; PTT, patellar tendon thickening; TT, tibial tuberosity.

*Group 2*: Records of 274 cases of TPLO were reviewed. Prior to exclusions, a physical review had been recommended for 53 cases, of which 8 did not attend (a non-adherence rate of 15%). Seventy-nine cases were subsequently excluded because of incomplete data, failure to meet the criteria for “uncomplicated TPLO,” or non-attendance at a recommended physical review, leaving 195 cases available for analysis. Active surveillance was carried out, and videos were created 4 to 14 weeks after TPLO, with a median of 7 weeks. The majority of videos (93.8%) were recorded 6 weeks or later after TPLO. Based on lameness of the operated leg observed in the video, surveillance questioning, or concerns reported by the owners, a physical review was recommended by the supervising veterinarian in 20% of the cases (*n* = 39). This took place 6–24 weeks after the operation (mean = 8.98, SD = 3.24, median = 8.00). Lameness of the operated limb was recorded in 20 dogs (51%) on physical examination. Radiographs of the operated limb were performed in all of these cases. Two additional dogs had radiographs performed due to contralateral cruciate ligament disease. Twelve of the dogs that underwent physical review were diagnosed with PT (6.15%). Radiographs had been performed on all of these dogs, and all but one had shown lameness during the physical review. In the dog without obvious lameness, direct palpation of the patellar tendon elicited a pain response. This indicates that 30.8% of the physically examined dogs and 6.15% of all cases were diagnosed with PT. This was significantly higher than in Group 1. Other owner-reported problems and abnormalities detected on physical examination and radiographs are summarized in Table 1.

The bias-corrected and accelerated (BCa) bootstrap method, based on 5,000 samples, estimated the proportion of PT diagnoses at 6.2%, with a 95% BCa confidence interval ranging from 3.6 to 8.7%.

## Discussion

The incidence of clinical patellar tendinitis after TPLO in a population of dogs followed up by RAS was assessed in 195 dogs. We found the incidence to be 6.15%, supporting our initial hypothesis and the premise that remote active surveillance may represent a viable alternative to standard in-person follow-up for the detection of clinically relevant patellar tendinitis after TPLO.

Radiographically, PTT is detected in the vast majority of dogs after TPLO (3, 9–11). In an earlier study, only dogs with severe PTT developed signs consistent with PT—7/94 dogs overall (~7%), which represented 29% (7/24) of those with severe thickening—supporting the concept that thickening alone is not synonymous with clinically relevant disease (10). The cause of and pathological differences between the lame and sound cases of PTT remain unknown and should be further investigated.

Active surveillance has been demonstrated to outperform passive follow-up for detecting surgical site infections in human and veterinary medicine (5–8). It has also proven valuable in oncology, where structured monitoring in low-risk cancers (e.g., prostate cancer and papillary thyroid microcarcinoma) enables timely detection of progression while avoiding overtreatment and preserving quality of life without compromising cancer-specific survival (21, 22). Using active surveillance, our detected incidence of PT was comparable to, or no less than, previously reported rates using traditional follow-up, and our bootstrap confidence intervals overlapped the 2–7% benchmark, indicating no lower detection rate with our method (3, 10). Bootstrap resampling repeatedly draws, with replacement, from the observed data to approximate the sampling distribution of a statistic (here, a proportion) and to obtain non-parametric, resampling-based confidence intervals; the BCa method further adjusts for bias and skewness (23). These preliminary findings suggest that the approach may be promising, although interpretation should remain cautious, as all available studies, including ours, were retrospective in nature and therefore reliant on the accuracy of existing medical records.

In our screening protocol, we implemented quality controls (including requesting repeat recordings of suboptimal videos) and adhered to published recommendations by assessing dogs outdoors at a walk and/or trot from the sagittal and frontal planes (24, 25). We mitigated the risk of potential underreporting bias through the use of active follow-up to identify any cases that might have otherwise been missed. By restricting inclusion to uncomplicated cases of TPLO, we aimed to reduce potential heterogeneity within the study population. Active surveillance in our study demonstrated improved adherence to follow-up compared with that reported previously (70% in 314 elective procedures) (4); however, some PT cases may still have been missed, as 15% of owners did not adhere to recommendations for an in-person review.

Two previous studies have analyzed the value of video assessment of gait to detect or grade lameness in dogs (26, 27). In one study, experienced observers repeatedly recognized obvious lameness on video; however, agreement with force plate measures declined once obvious trials were excluded, indicating that video assessment alone was unreliable for detecting subtle lameness (27). In another study, researchers found that video-based subjective gait analysis reliably differentiated lame from sound dogs with strong intra- and inter-rater agreement, and slow-motion assessment showed stronger, though not statistically significant, agreement with physical examination findings, particularly in identifying the affected limb (26). The findings from two further studies also indicate that subjective gait assessment of subtle lameness correlates poorly with objective measures such as kinetic or kinematic analysis, although agreement increased as lameness grade worsened (28, 29). Therefore, in the absence of objective gait analysis, standardized video review could serve as a practical, reproducible comparator to subjective gait analysis, adequate for distinguishing lame from sound dogs and identifying the affected limb.

Patellar tendinitis was chosen as the index complication because it is both common and clinically significant after TPLO (13) Because the majority of TPLO complications also declare themselves with lameness, PT could serve as a model complication for remote active surveillance that could be generalized to other events where lameness is the sentinel sign—for example, tibial tuberosity fracture, patellar fracture, and postoperative meniscal pathology or implant-related issues (30–32). This lameness-anchored framework prioritizes outcomes that matter clinically and are detectable without access to kinetic or kinematic instrumentation.

Group 1 was initially used to calculate the sample size required for Group 2, which represented the primary study group for which data were collected and evaluated. However, given that Group 1 represented a large dataset and, to the authors’ knowledge, the largest reported cohort of dogs available for the analysis of PT following TPLO in the current literature, it was deemed of interest to report these data alongside those of Group 2. A limitation inherent to Group 1 is that the dataset did not allow enumeration of all cases with PTT in that population, as only dogs in whom the management plan was altered at follow-up were systematically recorded—this may not have applied to all cases in which PTT was present, and a proportion may therefore have gone undetected or unreported. Furthermore, data on whether owners were adhering to the prescribed activity restriction protocol were not available for Group 1, which precludes any assessment of the potential influence of postoperative activity levels on the occurrence of patellar tendon thickening in this cohort. Additionally, demographic variables—including bodyweight, body condition score, and breed—were not formally compared between the groups, which represents a further limitation. However, given the considerable size of both cohorts, a degree of demographic heterogeneity is likely, which may partially mitigate the risk of systematic demographic bias between the two groups. Our preliminary findings demonstrated promise in the use of remote active surveillance, including video assessment, as a screening tool for detecting clinically relevant lameness, in this case attributed to patellar tendinitis after TPLO. Further prospective studies are needed to validate these findings, as, if validated, they could be of value to clinicians when making decisions regarding the follow-up of TPLO cases or when considering changes in protocol to increase workflow efficiency. Further research is also indicated to investigate the objective value of video recordings for lameness evaluation, as well as the factors that lead from PTT to lameness and pain. The high rate of adherence to follow-up underscores the value of active surveillance.

## Data Availability

The raw data supporting the conclusions of this article will be made available by the authors, without undue reservation. Requests to access these datasets should be directed to mmatiasovic@gmail.com.
